# Selenium Species in Diabetes Mellitus Type 2

**DOI:** 10.1007/s12011-023-03900-z

**Published:** 2023-10-25

**Authors:** Krystyna Pyrzynska, Aleksandra Sentkowska

**Affiliations:** 1 Faculty of Chemistry, University of Warsaw, Pasteur Str. 1, 02-093 Warsaw, Poland; 2 Heavy Ion Laboratory, University of Warsaw, Pasteur Str. 5A, 02-093 Warsaw, Poland

**Keywords:** Selenium species, Metabolism, Diabetes mellitus

## Abstract

Selenium is an important trace element for humans and animals as it plays a key role in several major metabolic pathways. Several studies were conducted to better understand the role of selenium against diabetes mellitus (DM), particularly type 2 (T2DM), but the obtained conclusions are contradictory. A simple linear relationship does not exist between the risk of T2DM and selenium levels but is best represented in a dose-dependent manner, getting often the *U*-graph. This relation also depends on selenium chemical forms that are present in a diet or supplements. Both too low and too high selenium intakes could increase the risk of diabetes. Moreover, the baseline status of Se should be taken into consideration to avoid over-supplementation. The focus of this brief overview is to report the recent updates concerning selenium participation in diabetes mellitus.

## Introduction

Selenium is an important trace element for humans and animals. It plays a key role in several major metabolic pathways such as antioxidant defence systems, thyroid hormone metabolism, DNA synthesis, and infection [1–3]. It is incorporated into selenoproteins as selenocysteine (the twenty-first genetically encoded amino acid), which is the main part of the antioxidant enzymes such as glutathione peroxidase and thioredoxin reductases [4]. Moreover, in populations with selenium low intake, its deficiency constitutes a risk factor for various forms of cancer and several serious diseases, including cardiovascular disease, diabetes, rheumatoid arthritis, nephropathy, asthma, and many others [5–8].

The Recommended Dietary Allowance (RDA) for adults is 55 to 70 µg per day based on the Se intake required to achieve maximum activity of glutathione peroxidase in plasma [9]. However, the Se amounts needed to reach maximal expression levels of other biologically important selenoproteins ascertainable in blood, such as selenoprotein P (SEEP1), may also be considered [10–13]. The maximum concentration of selenoprotein P in plasma was used as a criterion for the derivation of reference values for selenium intake in adults [11]. However, sexual dimorphism of SEPP1 is a biomarker of Se status in young subjects.

The European Food Safety Authority (EFSA) recommended lowering the tolerable upper intake level for selenium from 300 μg/day (proposed in 2000 by the European Commission) to 255 μg/day to reduce the risk of its adverse health effects [14] as higher levels of Se supplementation may have a negative impact [1, 15]. There is no sharp limit below which the deficiency of selenium occurs [10, 16]. Usually, this value is based on Se levels in a healthy population in a specific geographical area measured in blood plasma or serum [1, 9].

The diet’s main selenium sources are grains, bread, rice, meat, poultry, seafood, nuts, and eggs. Selenium bioavailability from the diet is affected by the composition of the food matrix and possible interactions (synergism or/and antagonism) among several components in a given foodstuff [17, 18]. Additionally, food processing could decrease its bioavailability. There is a large variability in the selenium content in foods, which depends to a large extent on the amount of selenium in the soil and on the capacity of a given plant to accumulate this element [19, 20]. Agricultural products in some regions (e.g., in Europe) are usually poor in selenium, if not supplemented via fertilizers or feed addition for animals. Several plants can accumulate Se inorganic species and transform them into selenoaminoacids. Selenomethionine (SeMet) is the dominant species in rice, while garlic, onion, or broccoli, vegetables from the *Allium* and *Brassicaceae* family, are better sources of *Se-*methylselenocysteine (MeSeCys) and γ-glutamyl-Se-methyl-selenocysteine (γ-Glu-MeSeCys).

Selenium exists naturally in the environment and accumulates in a variety of organisms as selenides (Se^2−^), elemental selenium (Se^0^), selenites (SeO_3_^2−^, Se(IV)), selenates (SeO_4_^2−^, Se (VI)), and in organic forms. These selenium species exhibit different chemical properties, biological utilizations, toxicity, and environmental effects. Generally, organic selenium species are considered more bioavailable for humans than inorganic forms, and Se (IV) is more toxic than Se (VI) [16]. It appeared that selenonanoparticles (SeNPs) are less toxic than inorganic and organic selenium based on their antioxidative activities and hematological parameters [21] and have a significant role in biomedical applications [22]. In addition, they are considered to be a better alternative for selenium supplementation due to improved delivery, absorption, and increased antioxidant capacity [23, 24].

Many reports indicate the association between low selenium intake with some chronic diseases. Se defends against free radical damage and inflammation and also plays an important role in maintaining a healthy metabolism [3, 9, 25, 26]. Because many selenoproteins participate in the cell antioxidative defence, Se was also suggested to play a protective role against diabetes mellitus type 2 (T2DM) [27–32]. T2DM is mainly characterized by high levels of glucose in the blood (termed hyperglycemia), deficiency of insulin secretion, or insulin resistance and may be caused by a combination of genetic or environmental factors. Too high a level of sugar in the blood can lead to serious health problems, such as heart disease, stroke, high blood pressure, and atherosclerosis [33–35]. The relationship between Se status and glucose control is not only limited to hyperglycemia but extends to hypoglycemia risk in Se deficiency as shown in the cross-sectional study of healthy subjects conducted in two regions in China with strongly different Se content in soil [36]. An important contributor to the development and progression of diabetes is the increase in oxidative stress.

The association between Se and diabetes mellitus is of great interest to researchers. Some of the reports indicate a link between certain selenoproteins involved in the insulin signaling pathway and insulin resistance and glucose metabolism [30, 31, 37, 38]. Several cross-sectional studies, case–control studies, cohort studies, and randomized trials were conducted to better understand the role of selenium against DM, but the obtained conclusions are contradictory [27, 29, 35]. It seems that the relationship between Se and T2DM seems to be more complicated [38, 39]. The focus of this brief overview is to report the recent updates (covering mostly 2017–2023 years) concerning selenium participation in diabetes mellitus. Interested readers could find more details concerning earlier contributions in the review papers [31, 40].

### Metabolism of Selenium Species

**Fig. 1:**
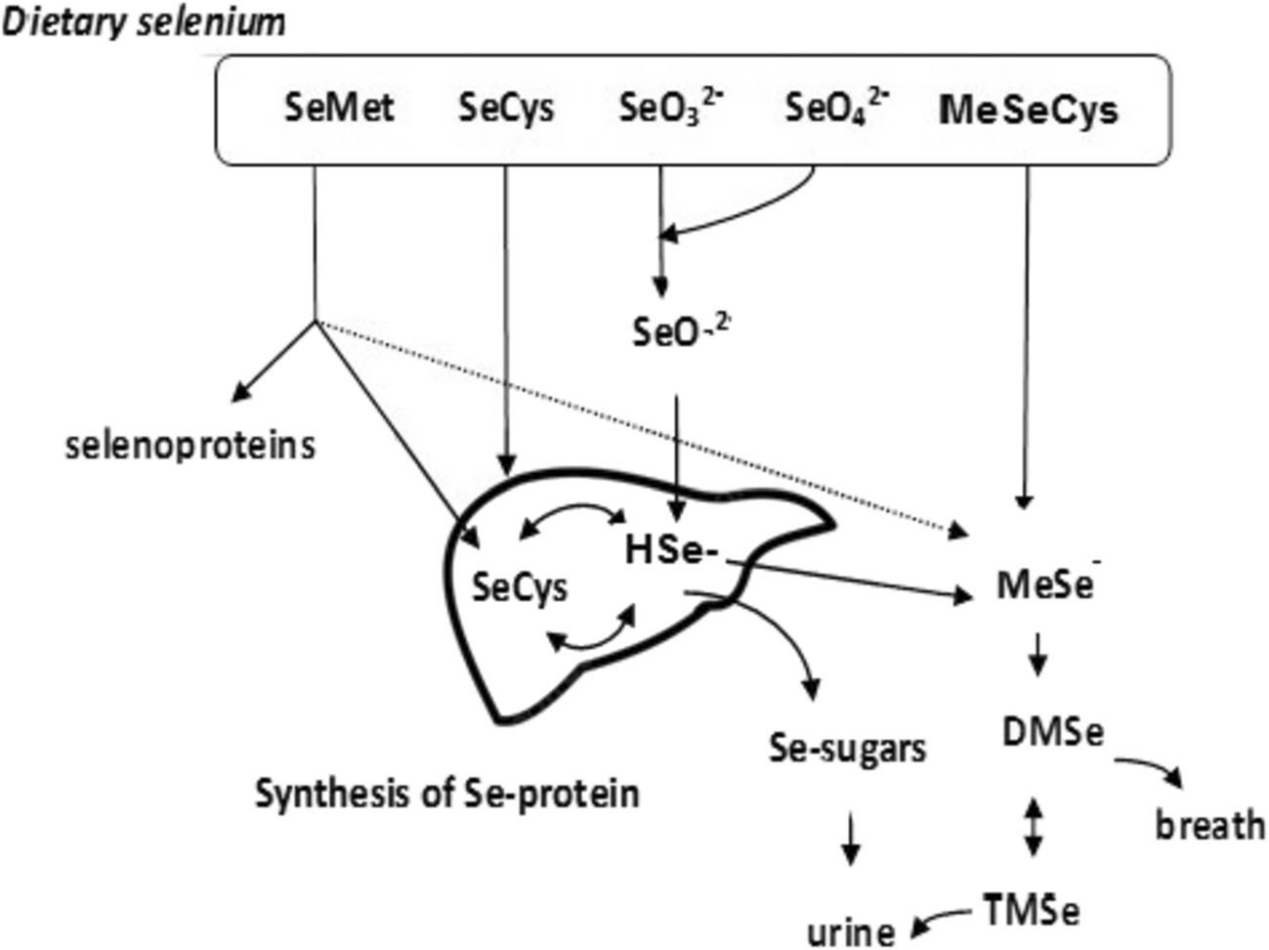
Schematic metabolic pathways of dietary selenium species. In enterocytes, SeMet and SeCys are absorbed by active transport, while selenate is absorbed by passive transport. After absorption, all forms of Se are converted to selenide through reactions that occur in the enterocyte. Part of SeMet is incorporated non-specifically in place of methionine into proteins. SeMet can also be transformed through selenocystathionine into SeCys, which is then converted to HSeˉ. In the liver, H_2_Se is converted to selenophospate and incorporated into Se-proteins in the form of SeCys residue by a unique UGA codon. Excess of selenium is excreted in the urine as selenosugars or TMSe ion as well as via respiratory track in the form of DMSe

Absorption of selenium ingested from diet and supplement sources takes place mainly in the small intestine and the retention of organic species is higher than that of its inorganic forms [41, 42]. Se metabolism is affected by its chemical form and by its specific or nonspecific incorporation into multiple proteins. Schematic metabolic pathways of dietary selenium species are presented in Fig. 1. Se (IV) in gastrointestinal fluid reacts with thiol groups of the reduced glutathione to form selenodiglutathione (GS-Se-SG), which is then reduced by glutathione reductase and NADPH to glutathione selenylsulfide and next to selenide. HSeˉ is in turn metabolized in the liver to form selenophosphate required for the further synthesis of Se-proteins. Selenoprotein P, synthesized and secreted by the liver with multiple selenocysteine residues, is a major selenoprotein in human plasma. It transports Se to several tissues, such as the brain and testis, to maintain antioxidative selenoenzymes, thus playing an essential role in selenium metabolism and antioxidative defence [49]. Se (VI) species, transported by diffusion through the intercellular space between the cells, can also be reduced to selenide by thioredoxin reductase/thioredoxin/NADPH system to HSeˉ [42, 43].

Dietary selenocysteine (SeCys) is found in animal products and after absorption by the digestive tract is carried to the liver, where is decomposed into selenide by the enzyme selenocysteine lyase (SCLY) [44]. Then, selenophosphate is synthesized from selenide by the selenophosphate synthetase 2 (SPS2) and is used to generate Sec-tRNA^Sec^. SeCys is incorporated into a polypeptide chain in response to a specific UGA codon using an intricate mechanism, that requires a hairpin steam-loop structure in *m*RNA called the selenocysteine insertion sequence (SECIS). MeSeCys, which is found in many vegetables, is directly methylated by β-lyases to MSeˉ, not to HSeˉ as in the case of other Se compounds [45]. It is worth mentioning that MSeˉ is contemplated as a key metabolite in the anticancer activity of selenium [46].

The part of selenomethionine transported to the liver can be non-specifically incorporated into proteins (e.g., albumin) in place of its analogue methionine. Some of these proteins can serve as an unregulated storage form of this element in the case of selenium deficiency [45]. SeMet can also be transformed through selenocystathionine into SeCys, which is then converted to HSeˉ [47]. Selenomethionine was also detected in the urine after ingestion, which suggests that to some extent it passes through the body in the unchanged form [48]. The detailed SeMet metabolism, the largest source of dietary selenium, was described by Gammelgaard et al. [49]. However, as it was reported, high selenomethionine supplementation can exhibit toxic effects [47, 50]. This compound can be transformed by metabolism to selenolates by GSH reduction, generating superoxide and hydroxyl radicals, potentially increasing the risk of cancer and CVD [50, 51]. A very high dose of selenium (300 μg/day, supplemented in the form of Se-yeast) dose of selenium, and taken for 5 years in a country with moderate to low Se status, increased all-cause mortality 10 years later [50]. However, such a phenomenon was not observed in the presence of selenocystine or selenocystamine [51].

The excretion of selenium from the human body can be realized in two possible ways. Under low-toxic Se status, HSeˉ or MSeˉ is transformed into selenosugars detected in urine [42]. At higher Se intake, it is removed from the body as volatile dimethyl selenide (DMSe) in the exhaled air or as trimethylselenonium ion (TMeSe^+^) in the urine. It should be added that urinary Se excretion appears to differ among males and females. It was reported that in a cohort of healthy, non-deficient Americans, the dose-dependent urinary Se excretion of women was 74% greater than that of men Se, even though both showed similar plasma Se responses to comparable intakes of Se [12].

The metabolism process of selenonanoparticles in the human body was very rarely studied as a part of Se species’ metabolic processes [23, 52]. It was not included in the proposed schematic metabolic pathways of dietary selenium [41, 42, 49, 53], although SeNPs are considered a novel Se supplement [24]. Kondaparthi et al. pointed out that nano-selenium is reduced by the thioredoxin (Trx) and glutathione (GSH) pathways that lead to the formation of selenide HSe ® anion [23]. Thus, H_2_Se is the precursor of selenoproteins for all nutritional sources of selenium and simultaneously the checkpoint intermediate for the utilization and excretion of this element.

### Selenium Species in Diabetes Mellitus

Generally, diabetes mellitus type 2 is characterized by hyperglycemia, insulin resistance (a hormone produced in the pancreas β cells that regulates blood sugar levels) and a relative decrease in its secretion. Along with T2DM development, pancreas increases insulin secretion to compensate for insulin resistance but later slowly fail to produce enough high amounts of insulin to control blood glucose [53]. Thus, high blood sugar occurs when a body either does not make enough insulin or cannot effectively use it. Maintaining proper blood sugar levels is crucial to the functioning brain, liver, and kidneys. Hyperinsulinemia, caused by insulin resistance, when the amount of insulin in the blood is higher than what is considered normal, is often associated with T2DM [54].

The protective effects of Se on DM are primarily attributed to its function in antioxidant defence. Selenium is not an actual antioxidant on its own but it is an integral part of selenocysteine residues. Thus, it plays a key role in forming the natural antioxidant enzymes involved in protecting against oxidative stress. Low expression or decreased activity of these enzymes, results in a large accumulation of reactive oxygen species that induces several cellular changes disturbing their normal physiological function. It has been also associated with an increased risk of certain metabolic disorders [55–57]. Oxidative stress not only promotes the onset of diabetes but also makes the disease condition worse [58].

### Animal Models

The animal models play an important role in exploring the mechanisms associated with type 2 diabetes. Several studies have evaluated the influence of Se (IV) intake on blood glucose and insulin levels as well as pancreatic function in animal models [59–61]. In recent years, the scientific focus has been switched towards selenium nanoparticles as they have impressive therapeutic effects due to better production of reactive oxygen species in a dose-dependent manner, compared to selenite [62, 63]. It has been observed that treatment with SeNPs (diameter range of 210–245 nm) milling with *Loranthus micranthus* leaves extract enhanced insulin levels and regulation of oxidant stress molecular markers in streptozotocin-diabetic rats [64]. Similar results (at the same Se dose level of 0.1 mg/kg body weight per day) were observed when rats were treated with SeNPs, SeO_2_, or their mixtures [65]. Blood glucose in diabetic rats significantly decreased after three weeks of treatment with SeNPs (136 ± 2.21 mg/dL) and SeO_2_ (113 ± 3.44 mg/dL) compared to the control group (265 ± 3.22 mg/dL). The lowest value was determined for a combination of these selenium species (108 ± 1.8 mg/dL). Simultaneously, blood insulin level was increased, but similar values of 5.04–5.60 μU/mL were obtained (3.90 ± 0.15 μU/mL for the control group).

**Fig. 2:**
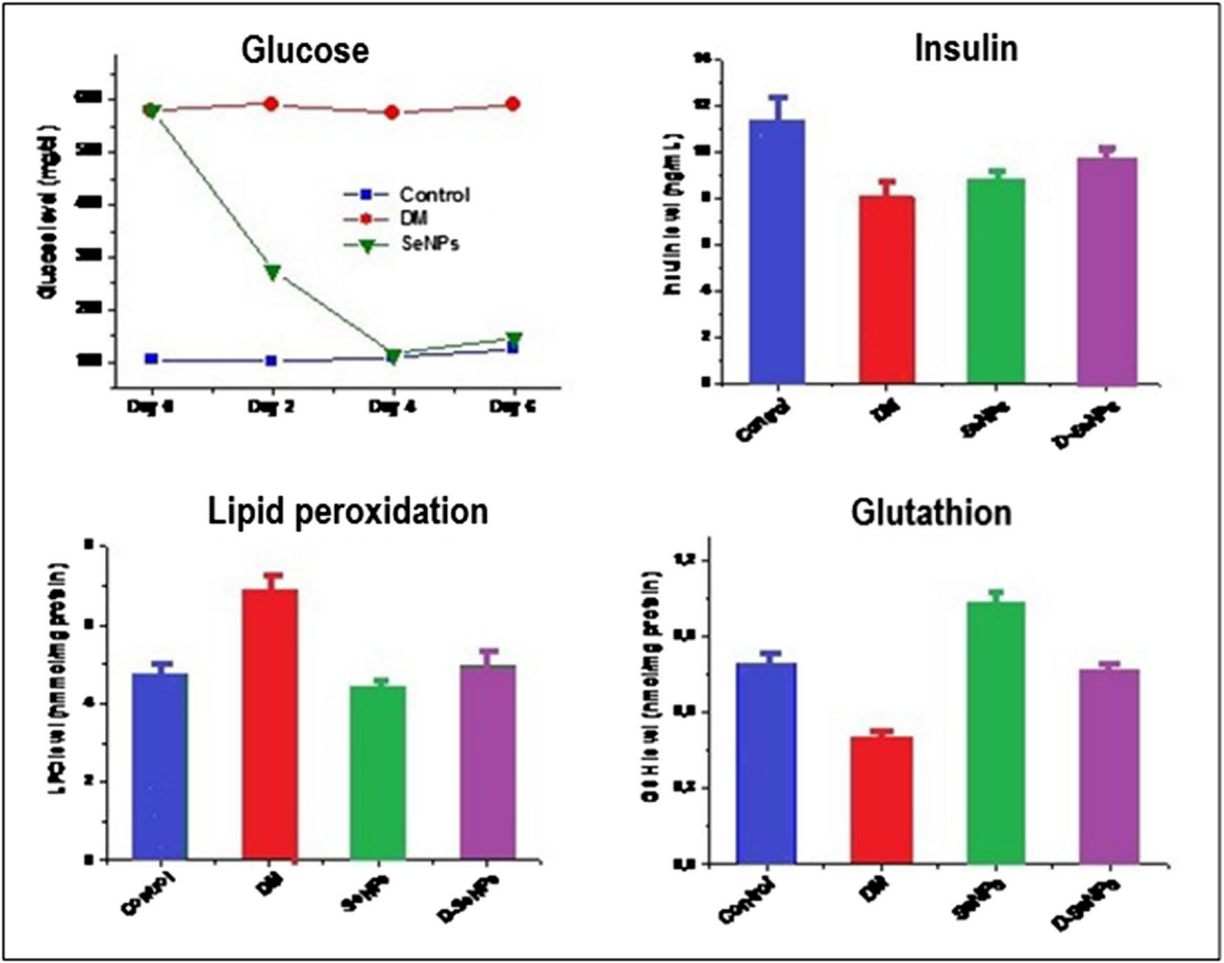
The plasma glucose, plasma insulin levels, and oxidative stress markers (lipid peroxidation, glutathione) of the control and tested diabetic rats were treated with SeNPs (0.5 mg) for 7 days. Abbreviations: DM, diabetes mellitus; SeNPs, SeNPs only; D-SeNPs, diabetic group treated with SeNPs. Reproduced under the CC BY license from reference [70]

The effect of SeNPs alone and in combination with the standard antidiabetic drug metformin (MET) was also examined [66, 67]. SeNPs combined with MET regulated the molecular markers of oxidant stress and tissue damage to greater excess than either SeNPs or MET alone. Other studies also indicated the antidiabetic and antioxidant effects of SeNPs of different sizes and administered in different doses [68–73]. Figure 2 shows the effect of SeNPs treatment on the plasma glucose and plasma insulin levels as well as oxidative stress markers (lipid peroxidation, glutathione) of the control and tested diabetic rats [70]. After administering SeNPs to diabetic rats, a prominent decrease in glucose levels was observed and the levels of plasma insulin were put back in the treated diabetic group. Some reports suggested that SeNPs exhibited their hypoglycemic properties by electing insulin–mimetic activity [69, 72, 73] like selenite [74] or selenate [75]. However, to induce insulin–mimetic activity, a very high dose of selenite was administered (0.2 g Se/kg), while the SeNPs dose was only 0.1 mg/kg.

It was demonstrated that vanadium compounds in animal experiments have several insulin-like effects and oral vanadium could be used in the therapy of diabetes mellitus [61, 76, 77]. El-Salami and Hanza evaluated the hypoglycaemic and antioxidant activity of VOSO_4_ and Se (IV) salt, either alone or in combination, against oxidative injury and hyperglycemia induced by experimental DM in rats [61]. The combination of vanadium and selenium species decreased blood glucose levels, elevated insulin hormones, pancreatic genotoxicity, and reduced oxidative stress to normal levels in comparison to the untreated diabetic group. However, no significant effect was found in blood glucose levels as compared with the control group. It should be noted that the long-term and/or chronic administration of vanadium compounds is related to its tissue significant accumulation and risks of toxicity [76, 77]. In the few studies on humans with positive results, vanadium compounds were administered only during very short periods [77].

The above-described works for animal studies reported that selenium species lowered blood glucose levels, raised the levels of insulin, preserved pancreatic β-cell function, and showed good antioxidant activity examined by the levels of oxidative stress markers in the liver and kidney. However, it has to be noted that some studies showed a negative association between selenium and the symptoms of type 2 diabetes [78]. The increased blood glucose levels and the decrease in pancreatic antioxidant capacity were observed when chickens were fed with a high dose of selenite (5.0 mg/kg) for 8 weeks, but the opposite results were obtained when a lower dose (3 mg/kg) was applied [59].

Animal models of T2DM that mimic human disease provide the opportunity to investigate the pathophysiology as well as evaluate potential strategies for treatment and related complications for humans [79]. However, human diabetes complications may occur over years, longer than the relative lifespan of most experimental animals. Thus, studies of insulin-deficient diabetes are often performed with levels of hyperglycemia over those found in even poorly controlled human diabetes. High Se dosage in the animal study (0.1 mg/day) for the adults would be extremely toxic in the long run [64].

### Human Studies

The association between selenium levels and diabetes mellitus is still discussed, either regarding its deficiency in humans or too much supplementation. The recent surveys have focused mainly on the correlations between type 2 diabetes mellitus and selenium intake [29, 34, 80–83], its level in plasma or serum blood [31, 37, 84–86], and Se metabolic comorbidities [87], and metabolic syndrome [88]. The association of selenium status with insulin resistance was also examined [6, 89, 90]. Several types of research observational and analytical studies have been used to estimate selenium safe dose and the risk of T2DM, each with its advantages and limitations [91, 92].

Cardoso et al. reported the results of the cross-sectional study which aimed to investigate the relationship between selenium status, and the markers of diabetes in a representative sample of the US population, considered to have moderate to high exposure to selenium [89]. The analysis included 4339 participants with age ≥ 18 years, from the National Health and Nutrition Examination Survey (NHANES, 2013–2018). The whole-blood Se concentration, fasting plasma insulin and glucose, glycohemoglobin (HbA1c), and homeostatic model assessment for insulin resistance (HOMA-IR) were measured. It was found that selenium status was positively related to insulin (*p*-value 0.003), glucose (0.037), and HOMA-IR (0.002) levels when the models were adjusted to age and sex. The additional adjustment to physical activity, smoking status, metabolic syndrome, and body mass index (BMI) showed the same association with insulin and HOMA-IR but was no longer significant with glucose (*p*-value 0.239). Based on these results, it was concluded the lack of dependency between Se and the prevalence of diabetes [89]. This conclusion counteracts the findings reported in another USA NHANES study (2011–2014, published in 2019) with 19,106 participants (age ≥ 20 years), adjusted for age, sex, race/ethnicity, BMI, hypertension, and dyslipidemia [30]. Fasting plasma concentration of glucose and serum Se were measured. This cross-sectional study showed that higher concentrations of Se were significantly associated with the prevalence of diabetes mellitus developing in a dose-dependent manner. In the multivariate logistic regression model, the increase of 10 μg/L in selenium increased the prevalence of DM by 12% (odds ratio (OR): 1.12; 95% confidence interval (CI): 1.06–1.18). As can be seen, both studies have some differences in the chosen covariates and metabolic syndrome–related factors.

The cross-sectional study conducted in Brazil (4106 participants only with high education) investigated the correlation between selenium dietary intake and T2DM [80]. This study evaluated its intake based on self-reported data on consumed food (as well as frequency and size) from the obtained questionnaires. On this basis, daily selenium consumption was calculated. The median energy-adjusted Se intake (evaluated to total energy intake) was 143.5 μg/day. No significant differences were observed between the participants with and without diabetes, but age, gender, sugar intake, and smoking differed (*p* < 0.05). The results of this study did not show a positive and significant correlation between energy-adjusted selenium consumption and the prevalence of T2DM [80]. Similar results were reported earlier comparing the appearance of T2DM between the control group and that which was daily supplemented with 200 μg of Se as selenised yeast [82]. The results of the cross-sectional population-based study on North Chinese adults (8824 adults, including 1804 with diabetes, aged 20–74 years) were reported by Siddiqi et al. [37] with relevant information, such as dietary habits, physical examination, lifestyle, as well as demographic measurements. The average nutritional intake of selenium was 52.43 μg per day, close to the recommended Se nutritional level (no users of Se supplements were detected). Fasting plasma insulin and glucose, HbA1c, HOMA-IR, homeostasis model assessment of cell function (HOMA-β), and oral glucose tolerance test (OGTT) were used as the markers of glucose metabolism. The odds ratio for developing diabetes mellitus was 1.66 (95% CI: 1.38, 1.99; *P* for linear trend < 0.005). Significantly, positive relations were observed between fasting blood glucose levels and HbA1c. It was suggested that the association between dietary Se and T2DM was potentially mediated by glucose metabolism (no statistically significant mediation effect was found for fast plasma insulin, HOMA-IR, and HOMA-β). In the earlier cross-sectional study among middle-aged and older adults in the central south area of China, a significant positive correlation between dietary selenium intake and the prevalence of diabetes was found [93].

The population-based cohort study in southern Italy was conducted to find the association between selenium intake and first hospitalization for T2DM with a median follow-up period of 8.2 years among 21,335 diabetes-free participants with their median value of daily Se intake equal to 59 μg [81]. During follow-up, 135 incident cases of hospital admissions for diabetes were indicated. The estimated hazard ratios (HRs) with 95% confidence intervals for diabetes hospitalization were 1.01 (0.60–1.70), 1.13 (0.66–1.96), and 1.75 (0.99–3.10) comparing second, third, and fourth sex-specific quartiles with the first quartile, respectively. HR level was 64% greater in the fourth quartile in comparison to the previous three. The main conclusion from this study is the positive association between dietary selenium intake above 60 mg/day and hospitalization for T2DM.

As conflicting results have been reported regarding the association of selenium with diabetes mellitus meta-analysis was applied to develop a more correct estimate of the effects’ magnitude [28, 31, 90, 93, 94]. That study design reduces bias and produces more reliable findings [95]. However, as it combines different types of studies, the summary effect may ignore important differences between studies. Both experimental and nonexperimental epidemiologic studies were examined regarding the Se dose–response relation with its serum and plasma levels using the spline regression meta-analysis [94]. The direct and roughly linear trend in that relation was found. Compared to the reference category of plasma or serum selenium levels (45 μg/L), exposures of 90 and 140 μg/L were associated with a ratio risk of 1.5 (95% CI, confidence interval 1.2–2.1) and 3.6 (95% CI, 1.4–9.4), respectively. Based on experimental studies with direct assessment of dietary selenium intake (supplemented mainly as selenised yeast), a similar increasing trend was observed. The intakes of 50 μg and 75 μg per day were associated with a RR of 1.5 (95% CI, range 1.1–1.9) and 1.9 (95% CI, 1.4–2.7) compared with the reference category (23 μg/day). The latter meta-analysis performed by Kim et al. showed that high concentrations of selenium are significantly associated with the presence of diabetes mellitus [28]. The odds ratio of 1.88 (95% CI, 1.44–2.45) was calculated, but with significant heterogeneity (*I*^2^ = 82%). In samples that used blood to estimate selenium level, a significant association was found with the presence of DM (OR 2.17; 95% CI, 1.60–2.93; *I*^2^ = 77%). Similar associations were for diet intake (OR, 1.61; 95% CI, 1.10 to 2.36; *I*^2^ = 0%), and urine (OR, 1.49; 95% CI, 1.02–2.17; *I*^2^ = 0%).

The meta-analysis aimed to identify the effects of selenium supplementation on insulin resistance and glucose homeostasis presented the opposite conclusion [90]. This study included 10 randomized controlled trials conducted in Iran and involving 526 participants with treatment periods between 4 and 24 weeks. Insulin levels, fasting plasma glucose, HOMA-IR, and HbA1c were defined as the primary outcome markers. Selenium was supplemented mainly as selenized yeast and sodium selenite (200 μg/day), but three studies did not mention the form of Se supplementation. The effect of selenium supplementation on relevant markers was assessed as the changes before and after treatment in the experimental and the control groups. The treatment dose (probably up to 200 μg/day) and period were not limited, as the authors reported. The current results suggested that selenium supplementation may be an effective treatment for reducing insulin resistance as it decreases serum insulin levels and HOMA-IR, but the effectiveness of Se supplementation on fast plasma glucose, levels was unclear.

**Fig. 3:**
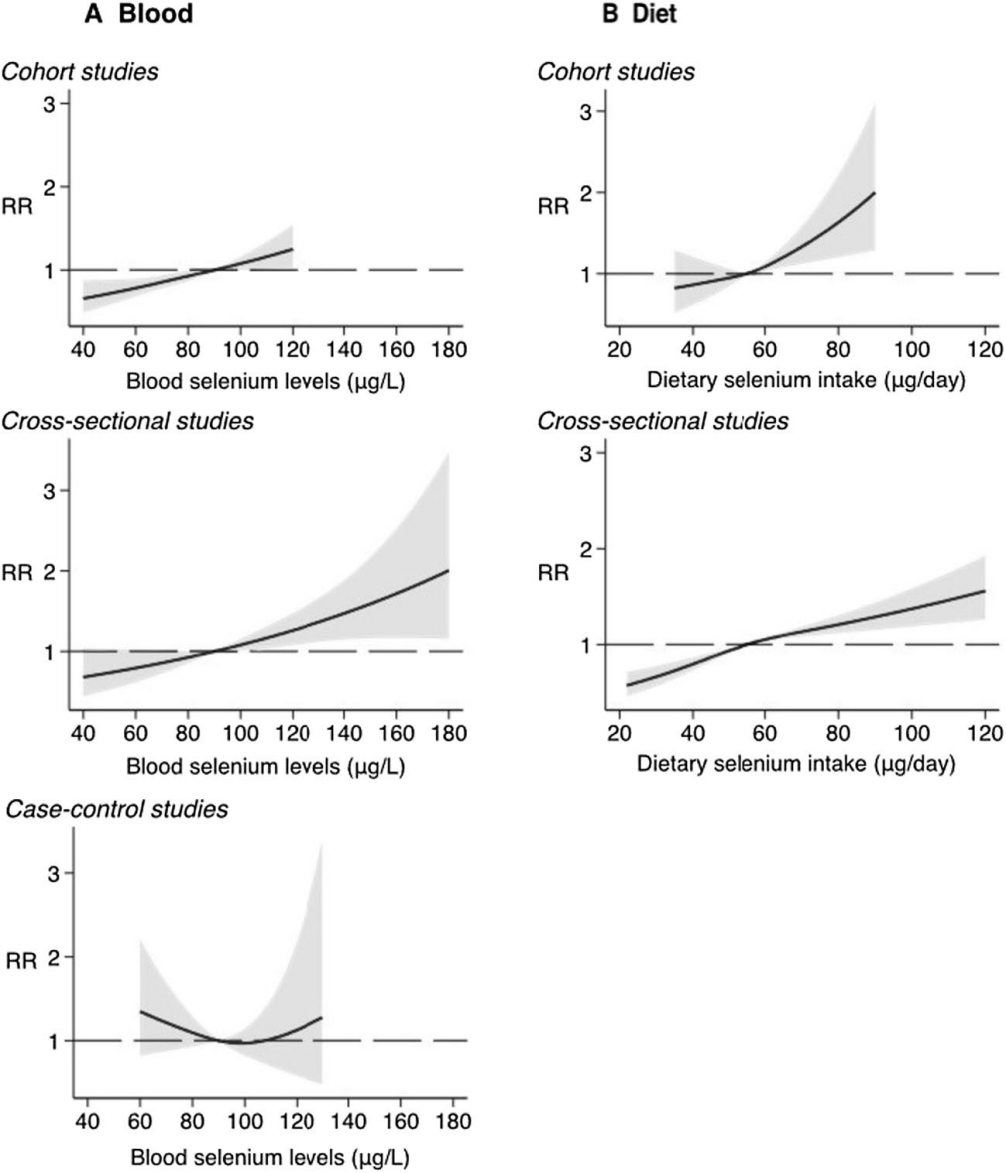
Dose–response association of **A** blood selenium concentration and **B** Se dietary intake estimated via food frequency questionnaire with the risk of diabetes (shaded area 95% confidence interval) in a one-stage restricted cubic spline model, according to study design. The risk ratio (RR) was based on 90 μg/L for blood selenium concentration and 55 μg/day for dietary selenium intake, as the referent category. Reproduced with permission from Elsevier reference [31]

Vinceti et al. presented the recent systematic review and dose–response analysis between selenium intake and the risk of T2DM according to the study design [31]. The examined cohort studies based on blood Se levels showed a positive effect on the risk of diabetes in the whole range of intake (Fig. 3A). This relation was also observed in the published cross-sectional studies. In contrast, for case–control studies, a *U*-shape association between blood Se and increasing risk was obtained with its lower value when selenium concentration was in the range of 60–100 μg/L. However, this effect was very small. In the meta-analysis based on the dietary selenium intake data, the risk ratio increased linearly across the entire exposure range (Fig. 3B). In both types of studies, there was a linear positive association for the dose-respond below 60 μg/L, and negative above that value. It should be mentioned that the dietary intake of Se was based on the questionaries from the patients and there is a lack of information regarding selenium chemical forms. From the obtained data, it was concluded that the increased risk of diabetes mellitus occurs when blood selenium concentration is equal to or greater than 120 μg/L; thus, it confirmed earlier findings [39, 94].

The meta-analysis of observational studies reported by Wang et al. included five studies conducted in the USA (2) and in Europe (3) with the participants varying from 445 to 8876 [95]. The association between serum selenium levels and T2DM also indicated a likely *U*-shaped non-linear dose–response relationship. Serum selenium levels were positively associated with T2DM in populations with relatively low serum selenium levels (< 97.5 μg/L) and those with high serum selenium levels (> 132.5 μg/L).

**Fig. 4:**
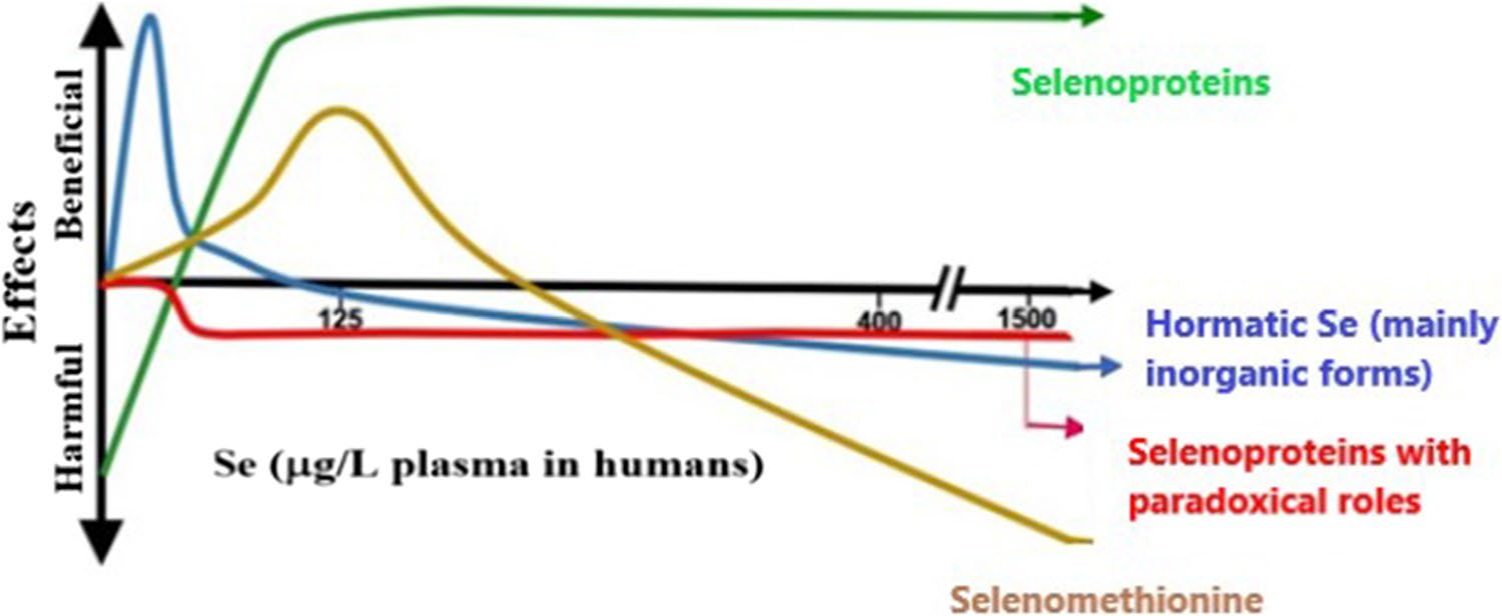
An illustration for the Se dose-dependent beneficial and harmful effects. This nutrient confers its impact on health through combined effects of nonspecific Se by stress-response hormesis and selenoproteins that can positively and/or negatively impact health depending on selenium intake. Reproduced under the CC BY license from reference [1]

The positive and negative effects of selenium depend not only on its dose but also on the chemical forms of Se that are present in a diet or supplements [1, 96, 97]. Inorganic selenium species exhibited mainly hormetic occurrence, which is characterized by a beneficial role at low doses and inhibition at excess as represented by the *U*-shape graph. SeMet at low doses can be metabolized to selenide and can act as a source of selenoproteins. But at a high dose, as was mentioned earlier, can undergo further metabolism and produce additional oxygen-reactive species, thus increasing the harmful effect of selenium [51, 52]. Generally, selenoproteins exhibit beneficial roles in the protection against redox imbalance, but some of them can play a paradoxical role, namely instead of protection, they cause a toxic effect. Excess selenoprotein P affects glucose metabolism [97], and the overproduction of GPX1 enzyme was deleterious for healthy mice with normal metabolic status [97]. Figure 4 presents the beneficial and harmful effects of selenium depending on its dose and its chemical form [1].

It should be noted that high glucose concentration is also a risk factor for many serious disorders and diabetes can cause complications in the functioning of many organs through multiple mechanisms [16, 87, 98–100]. Thus, selenium can be indirectly linked to a large variety of human health disorders, mostly by the role of its antioxidant enzymes in the reduction of oxidative stress. Common diabetes health complications include heart disease, nerve damage (neuropathy), chronic kidney disease (nephropathy), blocking the flow of blood (atherosclerosis), damage to blood vessels in the retina (retinopathy), and other problems with feet, oral health, and vision.

## Conclusions

The results of the recently published studies underlined the complex role of selenium in diabetes mellitus disease. The relationship between the levels of Se markers in blood or its consumption with the risk of diabetes mellitus is generally positive, but not always linear [30, 37, 59, 80, 84, 85, 89]. It depends on the kind of observational studies, the applied markers of selenium in the blood, the duration of Se supplementation, as well and the baseline Se status of the patients. However, some published reports postulated negative evidence regarding the high intake of Se or no association [78, 81, 83, 101]. The association of selenium with T2DM depends on whether selenium is to be used to prevent the disease or as a therapeutic agent. Moreover, differences in the geographical location of participants who participate in the research and their dietary Se intake can potentially affect the results. The trials of relatively high Se supplementation do not reflect the effects of its intake of foods. Additionally, in most cases, the level of selenium intake was calculated using dietary questionnaires.

The risk of diabetes mellitus is best represented in a wide dose-dependent manner, getting often the *U*-graph. It is easy to check that both too low and too high selenium intakes could increase the risk of diabetes. More Se is not necessarily better for reducing disease, but its baseline status should be considered to avoid over-supplementation. The need for extra Se is necessary only in regions where its levels are low. When the selenium serum or plasma level is about 125 μg/L, supplementation is unnecessary [1, 31]. It is worth mentioning that the *U*-shaped relationship between Se concentrations and all-cause or cardiovascular mortality in patients with hypertension [102] and some other diseases [103] was also noted.

Most human studies use SeMet or enriched-selenized yeast as supplements to study their action in preventing and delaying the progression of diabetes mellitus. The antidiabetic properties of SeNPs in treating diabetes and their role in T2DM were only evaluated in animal studies. SeNPs act as hypoglycemic agents by reducing oxidative stress, protecting the integrity of the β cells and enhancing insulin secretion, and blood sugar regulation [68–70, 72]. This new form of Se supplementation exhibits higher bioavailability and slow release, thus lowering the risk of its excess [24]. However, the SeNPs metabolism in human biological systems, the correlation with main selenoproteins, and their role in pharmacological protection should be studied more closely to understand their biological activities. Elemental selenium can form conjugate with proteins containing free thiol groups, which shows that elemental Se is not biologically inert and it may provide the basis of a new class of anticancer agents [104, 105]. It would be interesting to find if selenium nanoparticles have a similar *U*-shape dose-depend association with a risk of DM. Thus, in the future, addi6onal research is needed regarding potential differences in the toxicity profile of various selenium chemical forms.
